# Study on Rice Submergence Germination Through the Combination of RNA-Seq and Genome Resequencing Strategies

**DOI:** 10.3390/plants14193033

**Published:** 2025-09-30

**Authors:** Xin Wang, Feng Yu, Linfeng Feng, Mingdong Zhu, Pingfang Yang

**Affiliations:** 1 School of Life Science, Hubei University, Wuhan 430062, China; wangxin028@163.com (X.W.); yufeng@hubu.edu.cn (F.Y.); 18335855927@163.com (L.F.); 2 Modern Agriculture Innovation Institute, Hubei University, Wuhan 430062, China; 3 Stat Key Laboratory of Hybrid Rice, Hunan Academy of Agricultural Sciences, Changsha 410125, China

**Keywords:** submergence, coleoptile, amino sugar, nucleotide sugar, RNA-seq, resequencing

## Abstract

Submergence during germination is a major barrier to the adoption of direct-seeded rice (DSR). Despite its importance in overcoming this barrier, the genetic architecture underlying the rapid coleoptile elongation under submergence remains largely elusive. Through screening among 20 different rice cultivars, a submergence-tolerant cultivar Xian133 and a sensitive cultivar Chang15 were obtained. Comparative transcriptomics and whole-genome resequencing were conducted between these two cultivars. The results show that rapid germination under flooding is driven primarily by transcriptional reprogramming rather than by antagonistic gene regulation. Transcriptome-wide analyses revealed a significant enrichment of the amino sugar and nucleotide sugar metabolism pathway in tolerant cultivar. This was further supported by the fact that promoter variants at the key loci *OscPGM* and *OsAGPL1* modulate the expression of these genes and emerge as principal determinants of coleoptile elongation capacity under hypoxia. The identified single-nucleotide polymorphisms (SNPs) within these regulatory regions provide promising molecular targets for marker-assisted breeding of DSR cultivars.

## 1. Introduction

Rice (*Oryza sativa* L.), which feeds more than half of the world’s population, is highly vulnerable to various biotic and abiotic stresses. Among them, flooding is particularly devastating during the first two weeks after sowing. Because of the limited O_2_ and CO_2_ diffusion under water, aerobic respiration and photosynthesis are suppressed, which stalls reserve mobilization, and ultimately inhibits germination and early seedling growth. These early-stage injuries can result in a 10–70% yield losses, depending on flood duration and water depth [1,2]. The global climate change drives more frequent extreme rainfall events, which further limits the application of direct seeding technology and exacerbates the impact of submergence stress on seedling survival [1]. Under these conditions, the rapid elongation of coleoptile could function as a crucial escaping strategy that determines successful anaerobic germination and early seedling survival [3]. However, the genetic differentiation mechanism underlying coleoptile elongation ability among varieties has not been fully clarified, which hinders the breeding of rice cultivars with high ability of submergence germination. Compared with traditional transplanting, direct seeding offers clear advantages-reduced labor, lower production costs, and improved resource-use efficiency, making the elucidation of its genetic mechanisms an urgent breeding priority [4,5].

During long-term evolution, rice has evolved a sophisticated flood-adaptive system driven by metabolic reprogramming and hormone signaling networks. Starch is rapidly hydrolyzed, fermentation is activated, and energy and carbon skeletons are supplied for coleoptile elongation. α-Amylase [6], sucrose synthase [7], and trehalose-6-phosphate phosphatase TPP7 [8] are recognized as core determinants of anaerobic germination, and their efficient coupling is essential. Specifically, ethylene accumulation triggers a cascade of downstream signaling molecules (such as NO, ROS, Ca^2+^, and CO_2_), which jointly regulate key submergence tolerance mechanisms [9,10]. Ethylene, ABA, GA, and auxin form a multi-hormone cascade in which coleoptile polarity is controlled: the classic genes *Sub1A-1* [11], *ADH1*, and *PDC1* [12] are induced by ethylene, GA catabolism is inhibited, and elongation is promoted through the ethylene-*CIPK15* module [13]. ABA and JA activities are reduced by glycosylation via OsUGT75A [14], auxin is degraded through the SA-GH3 pathway mediated by OsCNL [15], ABA signaling and GA biosynthesis are balanced by OsGF14h [16], and promoter variation in OsCBL10 amplifies ethylene signals via the Ca^2+^-OsCIPK15 module [17]. Submergence tolerance is further enhanced by the qSHS5 haplotype through cell-cycle regulation and ROS homeostasis [18]. When recent GWAS and biparental QTL mapping results are integrated [19,20,21,22,23], a unified hormone–metabolism–signaling module is revealed, genetic divergence of coleoptile elongation is explained, and new gene resources and a theoretical framework are provided for molecular breeding.

Although the roles of ethylene signal transduction [24,25,26,27] and carbohydrate metabolism [28,29,30,31] have been partially elucidated, the function of the nucleotide sugar-mediated glycosyl donor synthesis pathway in regulating cell wall dynamics and polar coleoptile elongation remains unexplored. This pathway directly provides key precursors such as UDP-glucose and UDP-xylose, which are involved in the biosynthesis of cellulose, hemicellulose, and other polysaccharides. Its flux may determine the mechanical elongation capacity of the coleoptile and become a potential rate-limiting step in submergence adaptation. To genetically dissect this mechanism, we selected two contrasting indica varieties, the submergence-tolerant Xian133 and the submergence-sensitive Chang15, for comparative analysis. Although both belong to the indica subspecies, they represent distinct genetic backgrounds with markedly divergent profiles in submergence response. Kinship analysis based on genome-wide SNPs revealed an identity-by-state (IBS) value of –0.997 between them, indicating extremely low genetic relatedness and substantial allelic differentiation. This wide genetic divergence provides an ideal model system for pinpointing functional allelic variation underlying submergence tolerance, while minimizing background noise from incidental similarity. In this study, by comparing the differences in submergence stress responses between the submergence-tolerant variety Xian133 and the submergence-sensitive variety Chang15, and combining multi-time-point transcriptomic analysis with whole-genome resequencing, we have, for the first time, revealed the central role of the amino sugar and nucleotide sugar metabolism pathway in regulating coleoptile elongation.

## 2. Results

### 2.1. Screening of Rice Cultivars with Contrasting Submergence Germination Ability

To identify rice cultivars with contrasting germination ability under submergence stress, twenty rice varieties comprising nineteen indica cultivars provided by the Hunan Academy of Agricultural Sciences and one japonica standard variety were evaluated (Table S1). For each variety, 20 seeds were subjected to germination tests under aerobic and anaerobic conditions simulating deepwater submergence, with three independent biological replicates. Coleoptile length was measured on day 7 post-germination.

**Figure 1:**
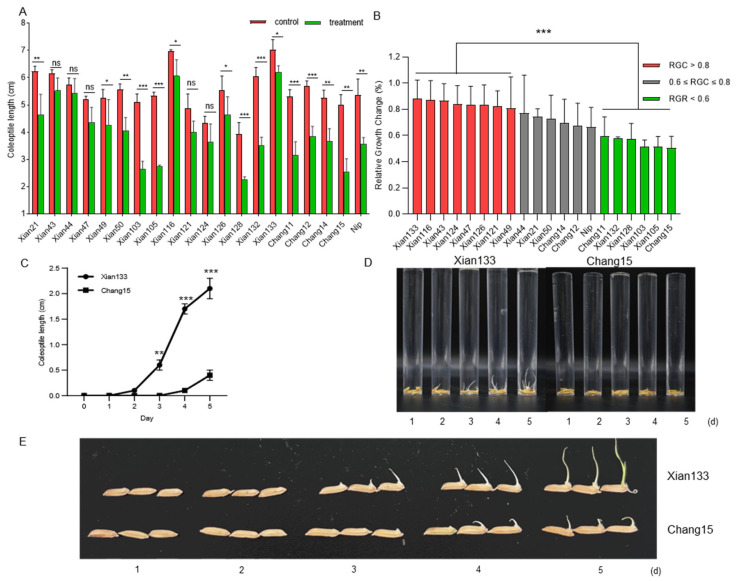
Phenotypic divergence in coleoptile elongation among rice varieties under submergence stress. (**A**) Variation in coleoptile elongation among rice varieties under control and deepwater stress conditions. Coleoptile lengths were measured on day 7 post-germination. Error bars indicate the standard deviation of three biological replicates. Different letters above the bars denote significant differences among varieties. (**B**) Relative growth change in coleoptile elongation under deepwater stress relative to control conditions for each rice variety. The relative growth change was calculated as the ratio of coleoptile length under deepwater stress to that under control conditions on day 7. Varieties are classified into three groups based on their relative growth change: red bars indicate high tolerance (relative growth change >0.8), gray bars represent intermediate tolerance (0.6 ≤ relative growth change ≤ 0.8), and green bars indicate low tolerance (relative growth change <0.6). Error bars indicate the standard deviation of three biological replicates. (**C**) Time-course analysis of coleoptile elongation in Xian133 and Chang15 under deepwater stress. Coleoptile lengths were measured daily from day 1 to day 5 post-germination. Error bars indicate the standard deviation of three biological replicates. Asterisks denote significant differences between Xian133 and Chang15 at each time point. (**D**) Growth status of Xian133/Chang15 at day 5 under 12 cm deep water in plastic cylinders. (**E**) Representative germination profiles of Xian133 and Chang15 under deepwater conditions. Coleoptile lengths of three representative seeds of each variety were measured daily from day 1 to day 5 post-germination. In (**A**–**C**) data were presented as mean ± SD (*n* = 3 biologically independent samples). Significant differences were determined by two-tailed Student’s *t*-tests (* *p* < 0.05, ** *p* < 0.01, *** *p* < 0.001). Source data are provided as a Source Data file.

The results showed that there are significant variations in coleoptile length among these varieties under both conditions, and anaerobic condition leaded to a decrease in coleoptile length in all the varieties (Figure 1A). To more accurately evaluate the effect of deepwater germination on the elongation of coleoptile among varieties, the relative growth change in coleoptile (coleoptile elongation under deepwater stress relative to control conditions) was calculated. Based on these data, Xian133 was identified as the variety with the strongest deepwater coleoptile elongation capacity, whereas Chang15 was the weakest one (Figure 1B). Given the extreme differences between Xian133 and Chang15, we further analyzed their coleoptile elongation dynamics during germination under anaerobic condition. The results showed that starting from day 3 post-germination, the coleoptile length of Xian133 was significantly longer than that of Chang15, and this difference increased over time (Figure 1C–E). These findings indicate a fundamental divergence in the coleoptile elongation ability of Xian133 and Chang15 in response to anaerobic germination conditions resulted by submergence, with this phenotypic differentiation detectable as early as day 3 and becoming more pronounced with time.

### 2.2. RNA-Seq Analyses on Xian133 and Chang15 During Submergence Germination

**Figure 2:**
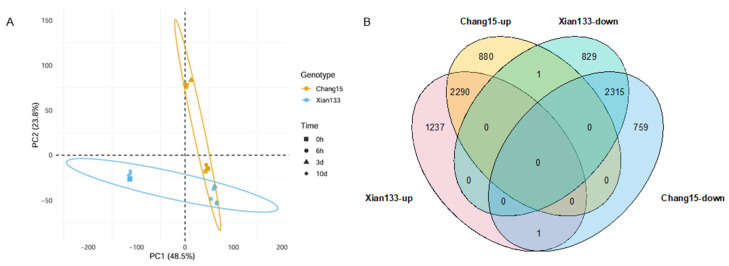
Transcriptomic overview and differential-expression dynamics. (**A**) PCA shows PC1 separates cultivars and PC2 follows the temporal gradient of submergence. (**B**) Venn diagrams illustrate the number and overlap of “persistently responsive” genes (consistently up- or down-regulated across all pairwise comparisons) in Xian133 and Chang15.

To explore the mechanism that mediates the difference in submergence germination, comparative transcriptomic analysis was conducted between Xian133 and Chang15 during submergence. lSamples were collected at 0, 6, 72 and 240 h of submergence germination, with three biological replicates per time point. RNA-seq yielded 24 libraries with an average of 47.98 million high-quality single-end reads per library (Q30 ≥ 94.57%, Table S2). PCA revealed that PC1 (48.5%) and PC2 (23.8%) together explained 72.3% of the total variance. PC1 clearly separated the two cultivars, whereas PC2 followed the temporal gradient, indicating that genotype predominantly shapes the global expression profile, while submergence duration drives the sequential responses (Figure 2A).

Differential expression analysis (FDR < 0.05 and |log_2_FC| ≥ 1) identified 2412, 3153, 1721 and 5823 differentially expressed genes (DEGs) at 0, 6, 72 and 240 h, respectively. Among these, 1100, 1371, 1177 and 2187 genes were up-regulated, and 1312, 1782, 544 and 3636 genes were down-regulated when compared Xian133 with Chang 15 (Figure S1). The DEG count peaked at 6 h, declined at 72 h and surged again at 240 h, suggesting an early, extensive transcriptional reprogramming followed by a transient stabilization and a final re-activation coinciding with the critical seed-survival phase.

We next defined “persistently responsive” genes that were consistently up- or down-regulated across all pairwise comparisons. Xian133 and Chang15 harbored 3529 and 3170 persistently up-regulated genes, respectively, with 2315 genes shared; 1237 and 829 genes were cultivar-specific (Tables S3 and S4). For down-regulated genes, 3191 (Xian133) and 3076 (Chang15) were persistent, including 2135 shared genes and 880 and 759 cultivar-specific genes (Tables S5 and S6), respectively (Figure 2B). These data provide a candidate gene set for further dissection of submergence tolerance differences between the two cultivars.

### 2.3. Functional Enrichment Reveals Divergent Transcriptional Strategies Underlying Coleoptile Elongation in Response to Submergence

**Figure 3:**
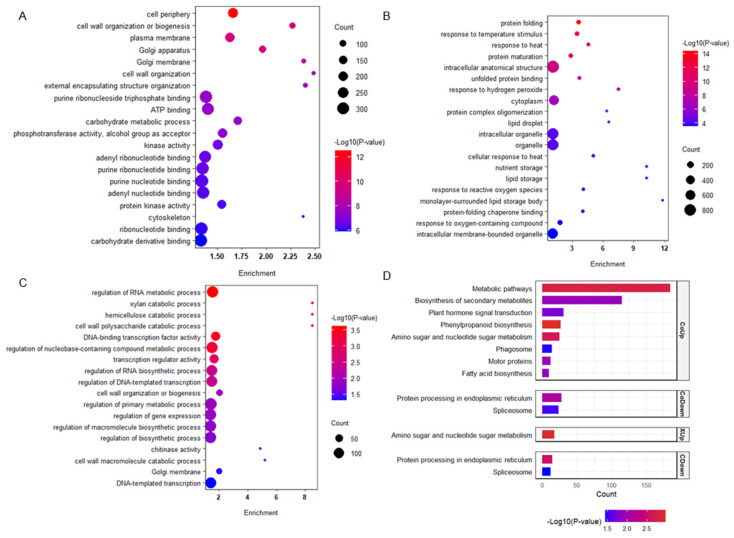
GO and KEGG enrichment profiles of differentially expressed genes under submergence. (**A**) GO terms enriched in genes up-regulated in both Xian133 and Chang15. (**B**) GO terms enriched in genes down-regulated in both genotypes. (**C**) GO terms enriched solely in Xian133-up-regulated genes. (**D**) KEGG pathways enriched in commonly up- and down-regulated gene sets. CoUp, genes up-regulated in both Xian133 and Chang15; CoDown, genes down-regulated in both genotypes; XUp, genes up-regulated only in Xian133; CDown, genes down-regulated only in Chang15.

To dissect the contribution of genotypespecific transcriptional reprogramming to the divergent elongation of coleoptiles under submergence, we performed GO and KEGG enrichment analyses on distinct gene sets. GO enrichment revealed that genes commonly up-regulated in both Xian133 and Chang15 were significantly enriched in cellular periphery (e.g., cell periphery, plasma membrane), cell-wall biogenesis and remodeling (cell wall organization or biogenesis, Golgi apparatus, Golgi membrane), as well as ATP-binding processes (Figure 3A). By contrast, commonly down-regulated genes were over-represented in protein folding and processing (protein folding, unfolded protein binding, protein maturation) and stress-responsive functions (response to temperature stimulus, response to heat) (Figure 3B).

Genotype-specific analyses further uncovered divergent strategies: genes uniquely up-regulated in Xian133 were enriched in transcriptional regulation (DNA-binding transcription factor activity, regulation of RNA metabolic process) and cell-wall metabolism (cell wall organization or biogenesis, chitinase activity) (Figure 3C). In Xian133, down-regulated genes enriched in nutrient reservoir, IgE binding, serine-type endopeptidase inhibitor, aleurone grain, intracellular structure, protein complex, transferase complex, and Cajal body indicate repression of storage, proteostasis, and organellar functions, consistent with metabolic re-programming toward energy conservation and stress adaptation (Figure S2). Conversely, Chang15-specific up-regulated genes were predominantly associated with DNA replication and cell-cycle progression (DNA replication, DNA replication initiation, nuclear origin of replication recognition complex, ADP binding) (Figure S3), implying a response to maintain meristematic activity and growth potential under submergence. Chang15-specific down-regulated genes were enriched in transmembrane transport of sugars and phosphoglycerates, as well as in RNA splicing (Figure S4).

KEGG profiling indicated that commonly up-regulated genes were enriched in phenylpropanoid biosynthesis, metabolic pathways, amino sugar and nucleotide sugar metabolism, biosynthesis of secondary metabolites, and plant hormone signal transduction, whereas commonly down-regulated genes were enriched in protein processing in the endoplasmic reticulum and the spliceosome pathway (Figure 3D). Notably, the amino sugar and nucleotide sugar metabolism pathway was significantly enriched in both the common up-regulated and the Xian133-specific up-regulated gene sets, underscoring its pivotal role in submergence responses and prompting us to pursue further investigation.

Collectively, these results reveal distinct transcriptional responses: Xian133 enhances cell-wall biosynthesis and remodeling to sustain elongation, whereas Chang15 activates DNA replication and cell-cycle processes but simultaneously represses transport and RNA metabolism, potentially limiting its adaptive capacity under submergence stress.

### 2.4. Amino Sugar Metabolism Pathway Key Gene Expression Patterns Validated Against Transcriptomic Data

To verify the reliability of the transcriptomic profiles and further elucidate the transcriptional regulatory mechanisms of the amino sugar and nucleotide sugar metabolism pathway (KEGG map00520) under submergence stress, we conducted quantitative real-time PCR (qRT-PCR) analysis on selected genes in both the tolerant Xian133 and sensitive Chang15 cultivars. This pathway was uniquely and significantly enriched in Xian133 under submergence (FDR < 0.01), and it synthesizes key nucleotide sugar donors—including UDP-glucose, UDP-galactose, and UDP-xylose—that drive cell wall remodeling and glycosylation modifications, potentially underlying differential coleoptile elongation between varieties.

The key genes involved in UDP-Glc and ADP-Glc biosynthesis (sub-pathway M00549), namely *OsHXK4* (*Os07g0197100*), *OscPGM* (*Os03g0735000*), *UGP1* (*Os09g0553200*), and *OsAGPL1* (*Os03g0735000*), were analyzed on their expression dynamics at 0, 6, 72, and 240 h post-submergence.

**Figure 4:**
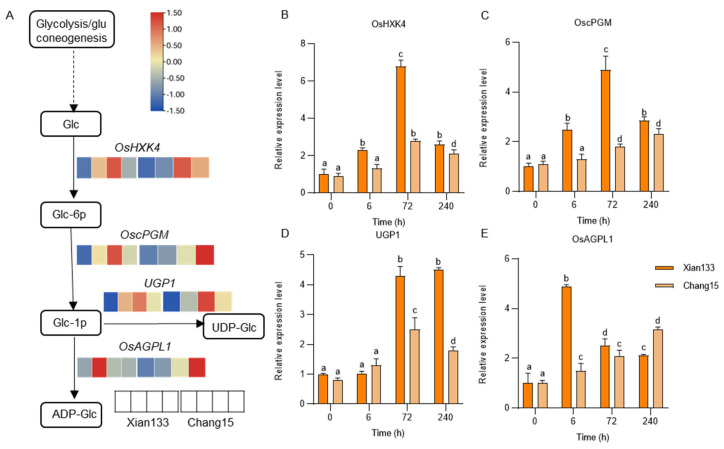
qRT-PCR validation of key genes in the amino sugar and nucleotide sugar metabolism pathway under submergence stress. (**A**) Schematic representation of the UDP-Glc and ADP-Glc biosynthesis branches within the amino sugar and nucleotide sugar metabolism pathway (KEGG map00520). The heatmap displays transcriptomic expression patterns of selected genes (*OsHXK4*, *OscPGM*, *UGP1*, and *OsAGPL1*) in the Xian133 and Chang15 under submergence. The color scale represents normalized expression levels, with red indicating high expression and blue indicating low expression. (**B**–**E**) Time-course expression profiles of *OsHXK4* (**B**), *OscPGM* (**C**), *UGP1* (**D**), and *OsAGPL1* (**E**) in both the tolerant Xian133 and sensitive Chang15 cultivars at 0, 6, 72, and 240 h post-submergence, as determined by qRT-PCR. Data are presented as mean ± SD (*n* = 3 biological replicates). Statistical significance was assessed using one-way ANOVA followed by Tukey’s HSD test. Different lowercase letters indicate significant differences (*p* < 0.05) among time points within each cultivar.

The qRT-PCR results were consistent with the transcriptomic data and revealed striking cultivar-specific differences. In Xian133, all four genes were significantly upregulated following submergence: *OsHXK4*, *OscPGM*, and *UGP1* peaked at 72 h, while *OsAGPL1* peaked at 6 h (Figure 4B–E). In contrast, Chang15 exhibited markedly lower expression levels of these genes across all time points, with minimal induction upon submergence. These findings confirm that the coordinated and sustained activation of the amino sugar metabolism pathway is a key mechanism underpinning enhanced cell wall synthesis and rapid coleoptile elongation in the tolerant Xian133 cultivar, while the weak response in Chang15 highlights a mechanistic basis for its sensitivity.

### 2.5. Genomic Landscape of Variation Between Xian133 and Chang15

Resequencing reads of Xian133 and Chang15 genomes were aligned to the Nipponbare reference. Xian133 yielded 185 million paired-end reads with 95.35% overall mapping and 90.87% properly paired reads; Chang15 produced 225 million reads achieving 97.21% mapping and 93.02% proper pairing. Both datasets exhibit uniform coverage and high quality, satisfying the requirements for downstream variant discovery.

**Figure 5:**
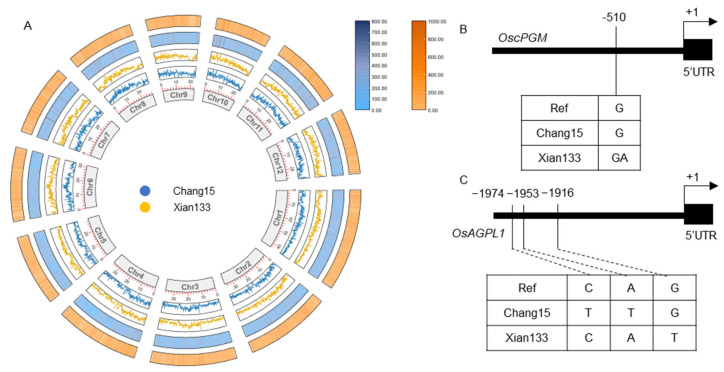
Genome-wide polymorphism landscape and promoter divergence of UDP-Glc and ADP-Glc biosynthesis pathways genes. (**A**) Circos visualization of variants between Xian133 and Chang15. Tracks from outermost to innermost: indel heat-maps (outer, Xian133; inner, Chang15) and SNP density line plots (outer, Xian133; inner, Chang15); centermost ring indicates the 12 chromosomes. A total of 5,590,317 SNPs and 931,335 indels were identified, 45.04% of SNPs and 43.08% of indels being cultivar-specific. (**B**) Detailed view of promoter SNPs in *OscPGM*. (**C**) Detailed view of promoter SNPs in *OsAGPL1*. Promoter regions were defined as the 2 Kb sequence immediately upstream of the transcription start site.

A five-layer Circos plot was constructed to visualize the genome-wide polymorphism landscape (Figure 5A). The innermost ring demarcates the 12 rice chromosomes. Two line tracks immediately outside display SNP densities for Chang15 (inner) and Xian133 (outer), followed by two heat-map tracks representing indel densities in the same order. In total, 5,590,317 SNPs and 931,335 indels were identified, of which 45.04% of SNPs and 43.08% of indels were cultivar-specific, underscoring pronounced genomic divergence between the two accessions.

We next examined four core genes of the UDP-Glc and ADP-Glc pathway. Since they are identified as DEGs, we just focused on their promoter sequence. Comparative analysis of promoter regions revealed cultivar-specific SNPs in *OscPGM* and *OsAGPL1* (Figure 5B,C). No sequence variants were detected in either the promoter or coding regions of *OsHXK4* and *UGP1* between the two cultivars. These promoter polymorphisms may modulate transcription-factor binding or chromatin accessibility, thereby contributing to the observed divergence in UDP-sugar biosynthesis and cell-wall remodeling under submergence stress.

## 3. Discussion

Rapid expansion of direct-seeded rice has rendered “the capacity to germinate under complete submergence” the foremost bottleneck in production. Although rice can survive temporary inundation, hypoxia markedly suppresses endosperm starch mobilization and coleoptile elongation, thereby hindering seedling establishment [32,33,34]. Deepwater rice circumvents this constraint by rapidly elongating internodes to keep leaves above the water surface, providing a classical paradigm for studying submergence tolerance [35,36]. These results are highly consistent with the findings in other plants tolerant to various abiotic stress, including salt-tolerant broccoli [37] and cold-acclimated freezing-tolerant woody plants [38]. Moreover, nucleotide sugars might also involve in plant biotic response [39]. Together, these studies demonstrate that maintaining the normal function of the amino sugar and nucleotide sugar metabolism pathway is a conserved strategy for plants to resist multiple stresses, providing a theoretical basis for improving crop multi-stress resistance by regulating this pathway.

Here, we selected two genotypes of rice exhibiting contrasting submergence tolerance phenotypes: the tolerant cultivar Xian133 and the sensitive one Chang15. We integrated transcriptome profiling with whole-genome resequencing to dissect their genetic basis. Phenotypic profiling of 20 rice accessions under deep-water stress identified the submergence-tolerant line Xian133 (longest coleoptile) and the sensitive cultivar Chang15 (shortest coleoptile) as the most contrasting pair (Figure 1). Strikingly, no classic antagonistic expression pattern was observed; genes up-regulated in Xian133 were not reciprocally down-regulated in Chang15, and vice versa (Figure 2), indicating that phenotypic divergence is unlikely governed by a simple on/off switch but rather by genotype-specific regulatory networks. Differential expression analysis revealed significant enrichment of the amino-sugar and nucleotide-sugar metabolism pathway among both shared and cultivar-specific up-regulated genes (Figure 3 and Figure 4). This pathway generates UDP-glucose and ADP-glucose, precursors for cell-wall polysaccharides and mediators of osmotic adjustment. The submergence-tolerant gene *OsUGT75A*, a member of this glycosyltransferase family, has been reported previously [14], suggesting that enhanced cell-wall expansion and osmoregulatory capacity accelerate coleoptile elongation. Notably, promoter regions of the key genes *OscPGM* and *OsAGPL1* harbored unique SNPs (Figure 5) that are predicted to alter transcription-factor binding or chromatin accessibility and thereby drive differential expression. Based on the RGC, we selected submergence-tolerant cultivars (Xian116 and Xian43, RGC > 0.8) and sensitive cultivars (Xian105 and Xian103, RGC < 0.6) and analyzed the expression dynamics of *OscPGM* and *OsAGPL1* at five time points under both aerobic and anaerobic conditions. qRT-PCR results demonstrated that under submergence stress, the expression of both genes was significantly and sustainably upregulated in the tolerant cultivars, whereas the sensitive cultivars exhibited markedly attenuated and delayed expression responses (Figure S5).

Collectively, this study is the first to link the amino sugar and nucleotide sugar metabolism pathway, promoter-specific variants, and submergence-tolerant germination in direct-seeded rice. These findings provide clear targets for functional validation and molecular-marker development. Future work employing transgenic complementation, promoter-activity assays, and association analyses in diverse populations will elucidate the biological functions and breeding value of these loci in rapid coleoptile elongation under hypoxia.

## 4. Materials and Methods

### 4.1. Rice Cultivars and Germination Assay

To investigate the coleoptile length of different rice varieties during deepwater germination, we cultivated seeds from 19 diverse cultivars and the NIP variety in cylindrical culture tubes filled with water to a depth of 12 cm. The seeds were fully submerged at the bottom. All experiments were conducted under identical conditions, with the coleoptile growth of seeds recorded and a statistical comparison performed on day 7 to evaluate significance. Each experiment was repeated three times, using a total of 15 seeds per replication for germination analysis. The seeds were cultured in a constant-temperature chamber with a light intensity of 300 μmol·m^−2^·s^−1^, a temperature of 28 °C, a photoperiod of 14 h light/10 h dark, and a humidity of 60%.

From the above experiments, the varieties Xian133 and Chang15 were selected for further phenotypic comparison. Coleoptile lengths were measured on 1, 2, 3, 4, and 5 d after cultivation, and statistical comparisons were conducted to assess the significance of differences between the two varieties.

### 4.2. Transcriptomic Analysis of Xian133 and Chang15

For transcriptomic analysis, 20 seeds of each variety (Xian133 and Chang15) were taken. The lemma and palea were carefully removed using forceps, and the seeds were cultivated in 12 cm deep water for 6 h, 3, and 10 d. Seeds treated with 0 h of submergence stress were collected as a control. At the time points of 0 h, 6 h, and 3 d, the embryos of germinating seeds were used for RNA extraction; at 10 d after germination, the fully developed coleoptile were used. All samples were immediately frozen in liquid nitrogen and ground into a fine powder. Three independent biological replicates were prepared for RNA sequencing (Novogene, Beijing, China). Raw reads from Illumina sequencing underwent quality control and cleaning before being aligned with the rice reference genome IRGSP-1.0. The featureCounts software (version 2.0.6) was used to count the aligned reads, and gene expression levels were quantified using transcripts per million (TPM). Differential expression analysis was performed using the DESeq2 package (version 1.44.0) to identify differentially expressed genes (DEGs) with an adjusted *p*-value < 0.01 and a fold change ≥2 or ≤0.5. Gene Ontology (GO) enrichment analysis was conducted using the g:Profiler (https://biit.cs.ut.ee/gprofiler/gost, accessed on 8 May 2025) online tool to elucidate the biological functions, molecular processes, and cellular components of the DEGs.

### 4.3. RNA Extraction and qRT-PCR

To study gene expression in germinating seeds, total RNA was extracted using a Total RNA Isolation Reagent (Biosharp, Beijing, China) from Xian133 and Chang15 seeds germinated in deep water at 0, 6, 72 and 240 h to verify the reliability of the genes obtained from the transcriptomic data. The RNA was reverse-transcribed into cDNA using the HiScript III RT SuperMix for qPCR (+gDNA wiper) (Vazyme, Nanjing, China). RT-qPCR was performed using a Bio-Rad (Hercules, CA, USA) CFX384 Real-Time PCR Detection System with SYBR Green and gene-specific primers (Table S3). Actin was used as an internal control. The reliability of each sample was tested in triplicate using the 2^−ΔΔCT^ method. The PCR protocol included an initial denaturation step at 95 °C for 5 min, followed by 40 cycles of denaturation at 95 °C for 10 s, annealing at 58 °C for 10 s, and extension at 72 °C for 15 s.

### 4.4. Resequencing and Variant Calling

Genomic DNA of two rice varieties (Xian133 and Chang15) was extracted from powdered leaf tissue and sequenced on an Illumina platform (Benagen, Wuhan, China). Reads were trimmed with fastp (version 0.20.1) to remove adapters and low-quality bases, mapped to the Nipponbare reference (MSU version 7.0) with BWA-MEM (version 0.7.17), and sorted/indexed with Samtools (version 1.10). PCR duplicates were marked with Picard (version 2.26.0). Single-nucleotide polymorphisms (SNPs) and small indels were identified using GATK HaplotypeCaller (version 4.2.2.0) in GVCF mode, then jointly genotyped and filtered (QD ≥ 2.0, FS ≤ 60.0, MQ ≥ 40.0). Variants were annotated and visualized with TBtools (version 2.351) to highlight differences in target gene regions.

## 5. Conclusions

Integrating phenotypic screens with transcriptomic and whole-genome data, we delineated a genetic framework for rapid coleoptile elongation of rice under deep-water stress. Elongation divergence arises from cultivar-specific transcriptional reprogramming, with the amino- and nucleotide-sugar pathway prominently enriched, implying roles in cell-wall loosening and osmotic adjustment. Promoter variants in *OscPGM* and *OsAGPL1* may modulate this pathway, offering clues to expression divergence. In contrast, the sensitive cultivar Chang15 preferentially activates cell-cycle genes while repressing sugar transport and RNA splicing, constraining overall adaptability. These results link the amino sugar and nucleotide sugar metabolism pathway to submergence tolerance in direct-seeded rice, provide candidate targets for marker-assisted breeding and genome editing, and support the development of high-yielding, flood-resilient varieties.

## Data Availability

The raw sequencing data have been submitted to the NCBI Sequence Read Archive (SRA) under the BioProject accession number PRJNA1295744.

## Supplementary Materials

The following supporting information can be downloaded at: https://www.mdpi.com/article/10.3390/plants14193033/s1, Figure S1: Bar plots showing the numbers of differentially expressed genes (DEGs) between Xian133 and Chang15 at 0, 6, 72 and 240 h of submergence; Figure S2: GO enrichment of genes down-regulated uniquely in Xian133; Figure S3: GO enrichment of genes up-regulated uniquely in Chang15; Figure S4: GO enrichment of genes down-regulated uniquely in Chang15; Figure S5: Differential expression of key genes in tolerant and sensitive rice cultivars under submergence; Table S1: Rice cultivars evaluated for germination under submergence stress; Table S2: Transcriptome sequencing data after statistical analysis; Table S3: Xian133-unique sustained up-regulated DEGs; Table S4: Xian133-unique sustained down-regulated DEGs; Table S5: Chang15-unique sustained up-regulated DEGs; Table S6: Chang15-unique sustained down-regulated DEGs; Table S7: The primer sequences for qRT-PCR.
