# Supplementary figures and images for: Study on Rice Submergence Germination Through the Combination of RNA-Seq and Genome Resequencing Strategies
Source: Plants (Basel). 2025 Sep 30;14(19):3033. doi: 10.3390/plants14193033 (PMC12526484; doi:10.3390/plants14193033)

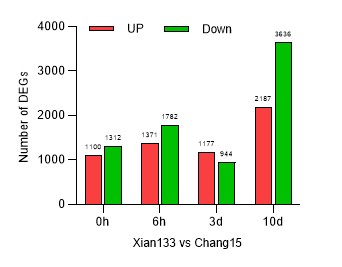

Supplement: Supplementary file 1 [file plants-14-03033-s001.zip › Figure S1.jpg]

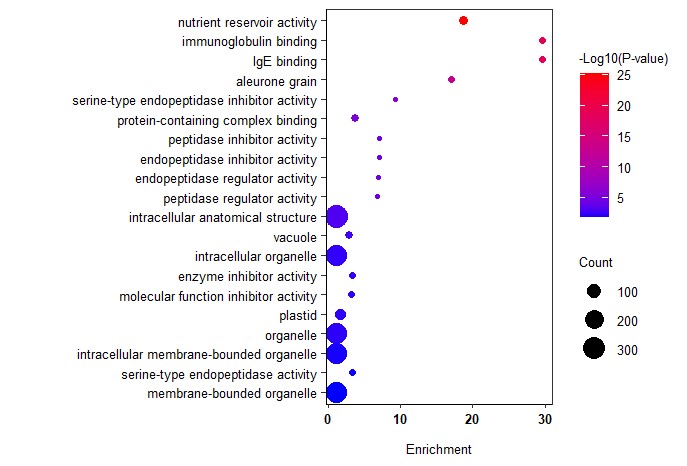

Supplement: Supplementary file 1 [file plants-14-03033-s001.zip › Figure S2.jpg]

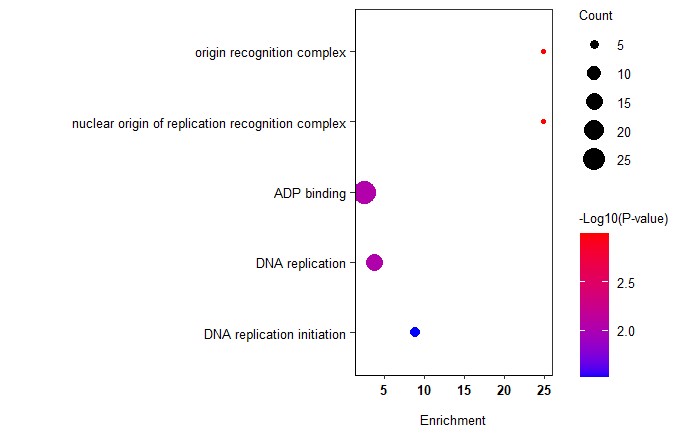

Supplement: Supplementary file 1 [file plants-14-03033-s001.zip › Figure S3.jpg]

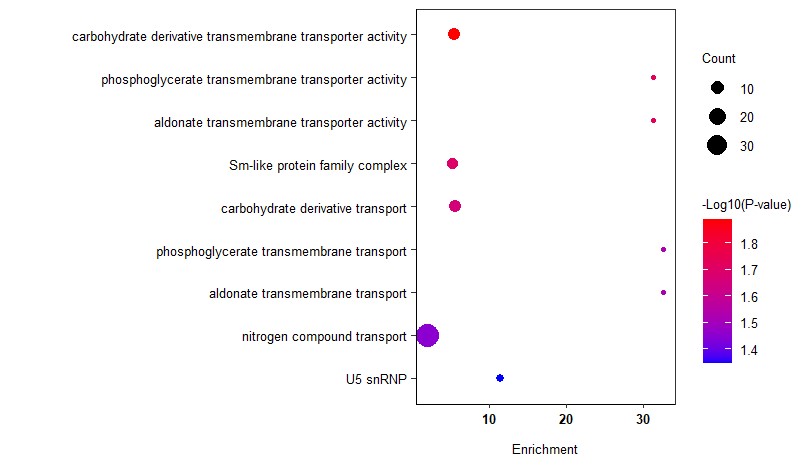

Supplement: Supplementary file 1 [file plants-14-03033-s001.zip › Figure S4.jpg]

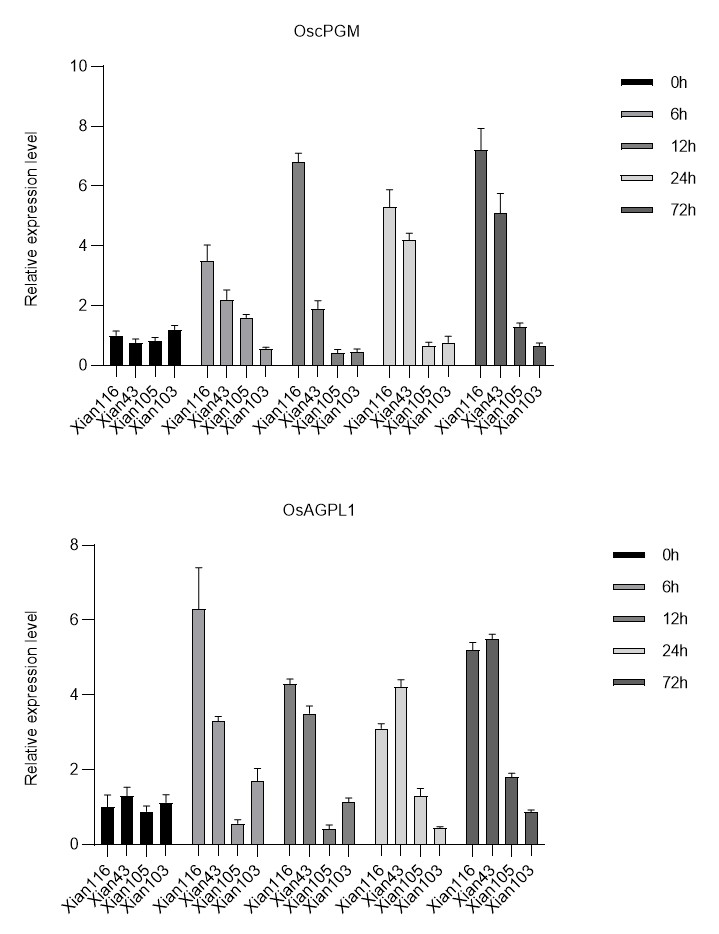

Supplement: Supplementary file 1 [file plants-14-03033-s001.zip › Figure S5.jpg]
